# A Rare Nasopharyngeal Hemangioma Presenting as Recurrent Cyanotic Apnea in a Neonate: A Case Report and Literature Review

**DOI:** 10.1155/carm/7094069

**Published:** 2025-09-27

**Authors:** Pershia Davoodi Karsalari, Samin Mortaheb, Heliya Rafsanjani, Noosha Samieefar, Aslan Ahmadi, Parinaz Alizadeh

**Affiliations:** ^1^Network of Interdisciplinarity in Neonates and Infants (NINI), Universal Scientific Education and Research Network (USERN), Tehran, Iran; ^2^Pediatric Urology and Regenerative Medicine Research Center, Gene, Cell and Tissue Research Institute, Children's Medical Center, Tehran University of Medical Sciences, Tehran, Iran; ^3^School of Medicine, Tehran University of Medical Sciences, Tehran, Iran; ^4^Pediatric Chronic Kidney Disease Research Center, Gene, Cell & Tissue Research Institute, Children's Medical Center, Tehran University of Medical Sciences, Tehran, Iran; ^5^Ear Nose Throat (ENT) and Head and Neck Research Center and Department, The Five Senses Health Institute, School of Medicine, Iran University of Medical Sciences, Tehran, Iran; ^6^Neonatal Health Research Center, Research Institute for Children's Health, Mofid Children's Hospital, Shahid Beheshti University of Medical Sciences, Tehran, Iran

**Keywords:** case report, cyanotic apnea, infantile hemangioma, nasopharyngeal mass, neonatal airway obstruction, premature infant

## Abstract

**Background:** Infantile hemangiomas (IHs) are the most common vascular tumors of infancy, but airway involvement is rare and potentially life-threatening. While subglottic and laryngeal regions are most frequently affected, nasopharyngeal hemangiomas are exceptionally uncommon, particularly in premature infants presenting with nonspecific symptoms such as apnea and cyanosis.

**Case Presentation:** We describe a female infant born prematurely at 35 weeks via cesarean section, who developed recurrent apnea and cyanotic episodes shortly after discharge. Flexible bronchoscopy revealed a nasopharyngeal mass causing upper airway obstruction, and imaging raised suspicion of a hemangioma. The patient underwent surgical excision of the lesion and was started on a tapering course of prednisolone. Postoperative recovery was favorable, and oral propranolol was initiated to prevent recurrence. At follow-up, the patient demonstrated normal development without respiratory symptoms.

**Conclusion:** Nasopharyngeal hemangiomas are rare and may present subtly in premature infants. Early bronchoscopy and imaging should be considered in cases of unexplained apnea or airway compromise. A multidisciplinary approach ensures timely diagnosis and effective treatment, minimizing long-term respiratory complications.

## 1. Introduction

Infantile hemangiomas (IHs) stand out as the prevailing vascular anomalies during infancy, typically emerging after birth [1]. Their life cycle unfolds uniquely, with rapid proliferation primarily within the initial 6 months of life, followed by a deceleration in growth, aligning with the child's development. Subsequently, they enter a phase marked by variable regression over subsequent years [2]. Vasculogenesis, the process by which new blood vessels form from angioblasts, drives the proliferation of IH. Hemangiomas may manifest in various body regions such as the skin, head and neck, internal organs, and limbs [3]. Recent studies estimate the overall prevalence of IHs at 2.8% among infants. Notably, nearly half of these lesions are found in the head and neck region, with a reported prevalence of 47.4% [4].

The occurrence of a large hemangioma in a fetus is uncommon, but fetuses presenting with large hemangiomas are susceptible to a variety of complications including heart failure, culminating in a poor prognosis situation [5]. These anomalies manifest superficially, deeply, or in a combined manner and fall into three morphological categories: solitary, segmental, or multifocal [1]. Airway IHs often coincide with segmental hemangiomas distributed in a pattern resembling a beard. As a result of small diameter of the airways in infants, the growth of IH can result in life-threatening obstructions, especially within the subglottic region, representing the narrowest segment of the infant airways. Therefore, early detection and adequate treatment strategies are paramount for the successful care [2, 6]. Despite numerous hypotheses regarding their origin, the precise etiology remains elusive, though compelling evidence points to their derivation from endothelial stem or progenitor cells [7]. While most are benign and resolve naturally over time, certain hemangiomas, depending on their size or location, can pose functional or life-threatening risks requiring immediate intervention [1, 8, 9]. In this article, we report a rare case of nasopharyngeal hemangioma, a rare but clinically significant involvement, in a premature infant experiencing episodes of cyanotic apnea.

## 2. Case Presentation

A two-month-old girl infant was referred to the emergency department of Mofid Children's Hospital, with cyanotic apnea. The patient was a preterm, delivered via cesarean section at 35-weeks' gestation, due to premature rupture of the membrane (PROM). The mother was a gravida 2 with a prior healthy 4-year-old girl.

**Figure 1:**
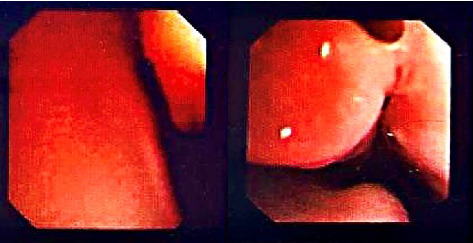
Endoscopic view of the posterior nasal cavity showing choanal stenosis observed during the initial bronchoscopy.

**Figure 2:**
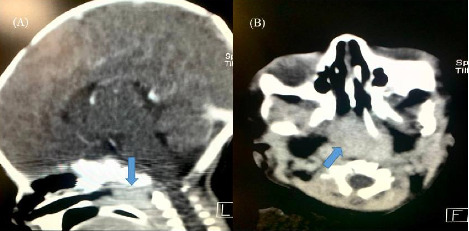
Spiral brain computed tomography (CT) scan. (A) Sagittal view and (B) axial (cranial) view. The arrows point to the enhanced adenoid region.

The preterm neonate, with a birth weight of 2600 grams (g), height of 49 centimeters (cm), and head circumference of 34 cm, was kept in the hospital for 5 days following delivery due to prematurity and was discharged without complications. However, postdischarge, the neonate experienced frequent episodes of apnea, specifically cyanotic apnea attacks, characterized by periodic occurrences lasting approximately 10–15 seconds (s). Subsequently, the patient was misdiagnosed with reflux by another physician, and treatment with pantoprazole was initiated until the age of 2 months. Regardless of that, as the duration of cyanotic apneic episodes lengthened, the patient was subsequently referred to Mofid Hospital, where the patient underwent intubation, during which no masses were observed, and the procedure was unremarkable in terms of difficulty. The mother reported that the cyanotic apneic episodes often occurred following crying spells. Later on, a cardiac consultation was sought to explore potential heart-related causes; but the cardiac evaluation yielded no abnormalities. Given the frequency of these episodes and their association with crying, a pulmonary consultation was pursued, leading to a bronchoscopy to investigate possible lower airway pathologies such as vascular external compression or other issues. During the bronchoscopy, narrowing of the choanal region was observed (Figure 1). While a mobile mass was suspected behind the choana, the findings were not definitive. This prompted consideration of various differential diagnoses, including choanal atresia or airway malacia. However, none of these conditions were confirmed during the procedure. Consequently, a spiral brain computed tomography (CT) scan with and without contrast was requested to characterize the mass composition further. During the CT scan without contrast, enhancement of the adenoid/nasopharyngeal region sparked inquiry into a multitude of diagnostic alternatives, including hemangioma, dermoid, lymphangioma, and other masses such as teratoma (see Figure 2).

**Figure 3:**
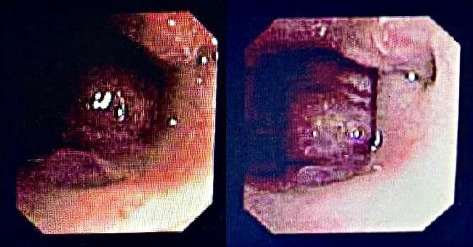
Perioperative bronchoscopic view demonstrating the obstructive lesion in nasopharynx.

**Figure 4:**
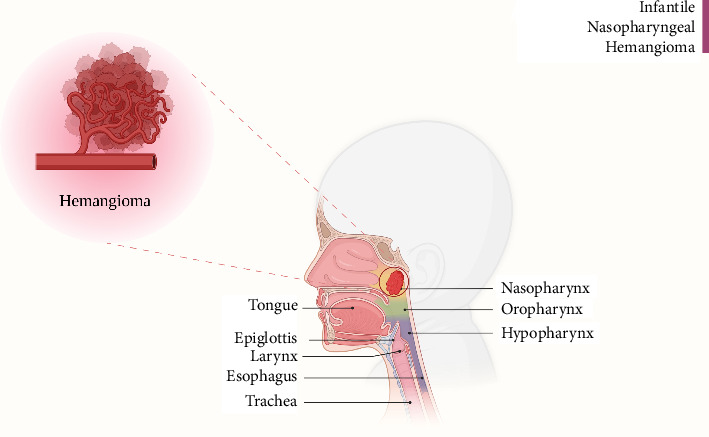
Schematic illustration showing the anatomical location of the mass. Created in BioRender. Davoodi Karsalari, P. (2025). https://BioRender.com/jt3z4nf.

**Figure 5:**
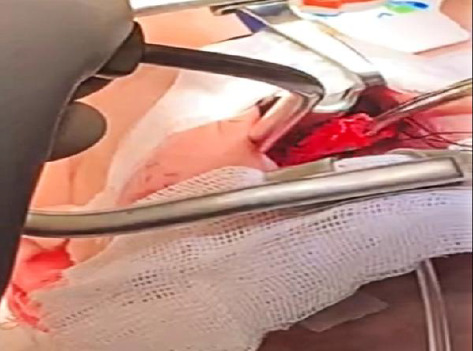
Intraoperative view showing transoral excision of the mass. A portion of the lesion is being removed, revealing its prominent vascular features.

The augmented mass, measuring 24 × 25 millimeters (mm), was detected, showing no signs of bone defect in the skull base. Due to its morphology and absence of cystic features, hemangioma emerged as the main diagnosis. Following the hemangioma suspicious, a subsequent bronchoscopy was performed, although a biopsy of the mass was not feasible. Notably, the vascular and sizable mass protruded from the child's mouth while in the hospital ward. An otolaryngology (ENT) consultation was sought, leading to a referral to Rasul Hospital. Before transfer, further investigations were conducted to rule out additional hemangiomas elsewhere in the body and viscera. Upon arrival at Rasul Hospital, another bronchoscopy revealed a mass extending the full length of the uvula (Figure 3). In line with the earlier bronchoscopy impression of the Mofid Hospital (Figure 1), attempts to approach the mass via the nasal route were unsuccessful as it ascended downward, necessitating a transoral approach. Following the transoral approach, attempts were made to fully excise the mass. Nevertheless, persistent hemorrhaging necessitated the division of the soft palate, revealing a vascular mass centered in the nasopharyngeal region with extension into the oropharynx, situated behind the uvula in the posterior soft palate. To better illustrate the mass, a schematic figure is provided to demonstrate its anatomical position (Figure 4). In Figure 5, the mass is shown being removed transorally, demonstrating its pronounced vascularity (Supporting Video 1). A thorough excision was performed, encompassing both the mass and the uvula, and the bleeding site was cauterized to achieve hemostasis. Subsequently, the soft palate was closed, and the patient was transferred to Mofid Hospital, where she remained intubated for 5 days. On the sixth day, massive hemorrhaging occurred through both the nose and mouth, leading to the patient's retransfer to Rasul Hospital. It was determined that the bleeding originated from the granulated tissue, likely resulting from the trauma of the tracheal tube insertion. After 2 days, another bronchoscopy revealed no complications, allowing for extubation. Under the guidance of an ENT specialist, treatment with prednisolone was initiated and later on scheduled to continue for 6 months, in addition to nystatin drops and propranolol. The patient was discharged and fully recovered without further complications.

## 3. Discussion

IH is commonly known as hemangioma of infancy and stands as the most prevalent tumor occurring during infancy, affecting approximately 2.8% of infants [4]. IHs tend to occur more frequently in females and are often located in the head and neck region. IHs are categorized into focal, multifocal, segmental, and indeterminate types based on their appearance and distribution and by the depth of the tissue affected as deep, superficial, or mixed [10]. The etiology of hemangiomas remains poorly understood. Their higher incidence in infants and females is yet to be definitively explained, although hormonal fluctuations are suspected. Multiple theories have been proposed, including involvement of angioblasts, trophoblasts, and dysregulation in cytokine-mediated pathways, which may trigger the angiogenesis. However, these theories remain not proven [11, 12].

In our case, a premature female infant experienced recurrent apnea and cyanosis following hospital discharge. Despite initial management for presumed reflux, the symptoms persisted, necessitating repeated intubations. This clinical course mirrors several reports in the literature, including those by Tamagno et al. [13] and Ţarcă et al. [14], where misdiagnosis as reflux or infection delayed recognition of an obstructive hemangioma. Bronchoscopy and imaging ultimately identified a nasopharyngeal mass obstructing the airway, which was subsequently excised. The patient was treated with oral prednisolone and propranolol, a regimen that has shown efficacy in multiple reported cases [15–17].

Airway endoscopy remains the gold standard both for diagnosis and follow-up, since it allows direct visualization of the region's location and extent of airway obstruction [18]. Flexible or rigid endoscopy enabled early diagnosis in many reviewed cases, such as those reported by Ajmi et al. [19], Kumar et al. [18], and Shellman et al. [20].

Our patient's CT scan revealed an enhancing mass in the nasopharyngeal region, raising concern for pathologic filling. Notably, adenoidal tissue is generally not visible on imaging in infants under 3 months [21]. Therefore, any soft tissue filling in this region observed in imaging of this age group needs further investigation as it may indicate the presence of mass or other pathological conditions [22]. As cited previously, a statistically significant association exists between prematurity and the development of IH in female infants [11], suggesting that these risk factors may have contributed to the presentation in our case. This correlation is supported by several reviewed cases, such as those reported by Tamagno et al. [13] and Ţarcă et al. [14], involving preterm female infants with subglottic or pharyngeal hemangiomas.

Infants presenting with stridor, cyanosis, or apnea are often misdiagnosed with viral infections or gastroesophageal reflux. This trend is evident in multiple cases we reviewed, including Choi et al. [23], Oliveira et al. [24], and Phipps et al. [25], where patients were initially treated for croup or reflux before a vascular tumor was identified. Temporary improvement with medications intended for viral etiologies may mask the underlying condition and delay definitive diagnosis [18]. The case by Kondamudi et al. in 2017 emphasized that recurrent apnea in preterm infants often has life-threatening causes requiring urgent evaluation [26]. Our findings underscore this point, aligning with the conclusions from Walner et al. [27] and Wang et al. [28] who emphasized early airway assessment in neonates with unexplained respiratory distress. Empirical treatment with pantoprazole for suspected reflux, as occurred in our case, carries potential risks and may delay life-saving interventions [29].

In conclusion, this case reinforces the importance of comprehensive evaluation in premature neonates presenting with apnea and cyanosis. Although rare, airway obstruction due to IH must be included in the differential diagnosis. Early recognition, accurate diagnosis via endoscopy, and prompt treatment, most effectively with propranolol, are crucial to prevent morbidity and mortality.

A consistent trend across the published literature is the high rate of delayed diagnosis due to misattribution of symptoms, particularly in cases without cutaneous markers. Furthermore, the diversity of anatomical involvement, from nasopharynx [1, 30] to subglottis [13, 21, 23] and even thyroid gland [26], emphasizes the need for high clinical suspicion across a range of presentations. Our findings support the growing consensus that propranolol is a safe and effective first-line therapy in most cases, aligning with outcomes reported in the recent literature. A summary of these previously published cases is provided in Table 1.

## Figures and Tables

**Table 1:** Summary of studies on infantile head and neck hemangiomas with respiratory manifestations.

Author/year	Study type	Age/sex	Previous medical history	Clinical manifestations	Imaging findings	Diagnosis	Treatment	Follow-up
Strauss et al. 1981 [15]	Case report	3 months old/male	Respiratory difficulty, grunting, and apneic spells during sleep within the first month of life, cutaneous hemangioma.	Breath holding during crying, chronic nasal congestion, cyanosis with apneic spells, coarse rhonchi from upper airway, feeding difficulty, weight loss.	Radiography and endoscopy detected an obstructive nasopharyngeal mass pushing the soft palate.	Nasopharyngeal capillary hemangioma	Prednisone	Significant decrease in lesion size
Ada et al. 2006 [16]	Case report	2 months old/male	Normal	Inspiratory stridor, dyspnea, normal cry, no feeding problem	Laryngoscopy demonstrated a soft red mass involving left arytenoid and aryepiglottic fold, further confirmed by biopsy.	Supraglottic hemangioma	Tracheostomy	Every 6-month follow-up with endoscopy revealed lesion disappearance by the age of 30 months
Ajmi et al. 2018 [19]	Case report	2 months old/female	Normal	Severe obstructive dyspnea, stridor, coarse respiratory sounds	Direct laryngoscopy demonstrated soft lesion closing 80% of the airway. Diagnosis was further confirmed by MRI.	Subglottic hemangioma	Tracheostomy, oral propranolol	Total regression of hemangioma at the age of 1 year. Although propranolol treatment continued until the age of 2
Azman 2022 [17]	Case report	3 months old/female	Normal	Admitted with severe croup and first diagnosed with laryngomalacia and subglottic edema. While after supraglottoplasty continued to have biphasic stridor.	Flexible laryngoscopy finally diagnosed hemangioma at the age of 7 months. Neck CECT also showed an enhancing mass at the right subglottic wall.	Subglottic hemangioma	Oral propranolol	Not mentioned
Choi et al. 2006 [23]	Case report	2 months old/female	Bronchiolitis with sustained stridor, cutaneous hemangioma of cheek, mandibular, and perioral areas.	Dyspnea, stridor	Bronchoscopy revealed swelling of the vocal cord and narrowness, axial CT showed an enhanced thickening of subglottic wall, and 3D VR revealed almost complete obstruction of trachea.	Submucosal subglottic hemangioma	Oral prednisolone	Marked regression of respiratory symptoms
Jacobson et al. 2014 [30]	Case report	Newborn (exact age was not mentioned)/male	Premature delivery (28 weeks), hyperbilirubinemia, respiratory distress syndrome	Biphasic stridor	Flexible nasopharyngolaryngoscopy revealed a supraglottic cause and bronchoscopy showed a deformation on the posterior tracheal wall obstructing 15% of the trachea.	Tracheal hemangioma	Propranolol	Lesion significant decrease in size after 8 weeks of treatment
Kumar et al. 2019 [18]	Case report	3 months old/female	Not mentioned	Severe respiratory distress, inspiratory stridor with moderate chest retraction. First diagnosed as croup due to cough, cold, and fever at admission.	Upper airway endoscopy revealed a mass on the right lateral subglottic wall with 70% occlusion of the airway. Contrast-enhanced CT also showed a well-enhanced lesion in the submucosal region of subglottic area.	Subglottic hemangioma	Oral propranolol	No recurrence of symptoms in the following 6 months after treatment. Normal development.
Leboulanger et al. 2010 [31]	Case series (retrospectively)	14 patients with mean age of 2.3 months at diagnosis/13 of them female	Two patients had posterior fossa malformations, hemangioma, arterial anomalies, cardiac anomalies, eye anomalies, and sternal anomalies (PHACES) syndrome. One patient had multiple cutaneous hemangioma. Also, two patients had severe reflux.	Not mentioned	Endoscopy revealed a mean subglottic occlusion of 68%. Pharyngeal and supraglottic involvement was also observed in two patients.	Subglottic hemangioma	Propranolol (11 patients had previously received steroids)	Significant reduction of respiratory symptoms. The obstruction reduced to 22% after 2 weeks and to 12% after 4 weeks.
Liang et al. 2020 [32]	Case report	2 months old/female	Normal	Laryngeal stridor, dyspnea, coarse breath sounds without rales	Ultrasonography demonstrated hypoechoic at the lower pole of thyroid. CT also revealed enlargement, multiple nodules, and inhomogeneous enhancement of parenchyma in the thyroid gland.	Thyroid capillary hemangioma	Surgery (total resection of left thyroid gland), oral propranolol	Normal thyroid function and no recurrence of hemangioma during 6 months follow-up
Oliveira et al. 2017 [24]	Case report	12 months old/male	Large posterior cervical hemangioma, stridor and respiratory distress since the first month, repeatedly misdiagnosed as croup.	Biphasic stridor becoming worse by agitation or supine position	Flexible bronchoscopy and MRI showed angiomatous malformation in subglottic region with more than 70% obstruction.	Multilobulated subglottic hemangioma	Injectional steroids, laser therapy (at the age of 5)	Normal growth and development
Onder et al. 2019 [33]	Case report	2 months old/female	Not mentioned	Respiratory distress, biphasic stridor, no feeding difficulty	Direct laryngoscopy and bronchoscopy revealed subglottic and aryepiglottic folds multiple hemangiomas. Chest and neck CT also confirmed mediastinal hemangioma compressing trachea.	Subglottic and mediastinal hemangioma	Oral propranolol	One year follow-up revealed significant decrease in the size of hemangioma.
Phipps et al. 1997 [25]	Case report (two cases)	Case 1: 2.5 months/female Case 2: 2 months/male	Case 1: Upper respiratory infection with stridor at the age of 1 month. First treated for gastroesophageal reflux based on her history. Case 2: Normal	Case 1: Stridor Case 2: Moderate retractions, biphasic stridor exacerbating with agitation or crying.	Case 1: Bronchoscopy demonstrated a soft red mass on the left subglottic side with 85% obstruction. Case 2: Direct laryngoscopy and bronchoscopy revealed a compressible mass covered with mucus located below the left true vocal cord.	Subglottic hemangioma	Case 1: Tracheotomy, surgical excision Case 2: Surgical excision	Both extubated on postoperative Day 3 and remained asymptomatic for 9–13 months of follow-up.
Robinson et al. 2021 [34]	Case report	4 months old/male	RSV bronchiolitis and hypoxemia	Brisk bleeding from left nare, oropharynx was also bloody without obvious source for bleeding.	Nasopharyngoscopy noticed a swelling in the left nare suspected to be the source of bleeding. While laryngoscopy showed a vascular mass in the epiglottis. MRI suggested the lesion as hemangioma or teratoma which was confirmed as hemangioma by biopsy.	Epiglottic hemangioma	Transfusion, steroids, propranolol, surgical excision	No complication during 6-month follow-up.
Shellman et al. 2023 [20]	Case report	Not mentioned/female	Cutaneous hemangioma on her upper back, biphasic wheeze which was misdiagnosed as new-born wheeze and chest infection.	Respiratory distress, failure to thrive, subcostal recession, biphasic stridor	Laryngotracheobronscopy revealed a large subglottic hemangioma with 80% occlusion.	Subglottic hemangioma	Propranolol (+dexamethasone)	Both cutaneous and subglottic hemangioma decreased in size after 1 week of treatment.
Tamagno et al. 2011 [13]	Case report	6 months old/female	Preterm, previously misdiagnosed as gastroesophageal reflux and recurrent respiratory infection.	Laryngeal stridor	CT scan demonstrated a vascularized heterogenous mass in left hemithorax and rigid bronchoscopy showed 80% obstruction of larynx and trachea.	Subglottic and mediastinal hemangioma	Propranolol (there was no clinical improvement with previous corticosteroid therapy)	Decrease in the size of lesion to less than 20% obstruction after 3 months of treatment.
Ţarcă et al. 2019 [14]	Case report	3 months old/female	Preterm, twin, low birth rate.	Acute respiratory failure, dysphagia, ‘wine stain' hemangioma located between eyebrows and frontal areas.	A purplish-blue mass was observed on physical examination in soft palate and lateral wall of pharynx. CT scan further demonstrated its location at the base of the tongue with an invasion to epiglottis and a significant effect on right tensor muscle.	Palatine velum and lateral pharynx wall capillary hemangioma	Oral propranolol	Significant decrease in size of genial and palatine hemangioma 4 months after treatment.
Walner et al. 2008 [27]	Case series (4 neonates)	First 10 days of life (2 female—2 male)	Abnormal Apgar score, two of them delivered with meconium stained amniotic fluid.	Respiratory distress, stridor, cyanotic spells, substernal retractions	Flexible laryngoscopy revealed a mass obstructing the glottic inlet by 50%–80%.	Lobular capillary hemangioma of larynx	Surgical remove by microlaryngoscopy (+corticosteroid +anti reflux medication)	No recurrence observed during follow-up
Wang et al. 2015 [28]	Case series (4 patients)	1 or 2 months old/all females	Not mentioned	Stridor, respiratory distress, tachycardia	Direct laryngoscopy showed vascular lesion with 50%–90% obstruction.	Subglottic hemangioma	Oral propranolol	No recurrence
Appiah-Thompson et al. 2023 [35]	Case report	8 days old/female	A fleshy swelling protruding out of the mouth periodically by crying.	Respiratory distress, feeding difficulty	Endoscopy of pharynx and larynx demonstrated a pedunculated mass attached to the left arytenoid by a stalk. Postoperative CT scan showed a poorly differentiated mass in left nasopharynx.	Laryngeal cavernous hemangioma	Surgical excision	Normal development in 7-month follow-up after operation.

## Data Availability

Data sharing is not applicable to this article as no datasets were generated or analyzed during the current study.

## Patient Perspective

The family was relieved once the accurate diagnosis and effective treatment were initiated, and they appreciated the dedication of the multidisciplinary care team in finally resolving the persistent symptoms.

## Supporting Information

Supporting information has been submitted along with this manuscript, including a completed CARE checklist and an intraoperative video demonstrating transoral excision of the mass (Supporting Video 1).
