# Supplementary material for: A Rare Nasopharyngeal Hemangioma Presenting as Recurrent Cyanotic Apnea in a Neonate: A Case Report and Literature Review
Source: Case Rep Med. 2025 Sep 27;2025:7094069. doi: 10.1155/carm/7094069 (PMC12496156; doi:10.1155/carm/7094069)
Supplement: Supporting Information — Additional supporting information can be found online in the Supporting Information section. [file 7094069.f1.zip › CARE Checklist- Infantile Hemangioma.docx]

Table 1. CARE Checklist of information to include when writing a case report

| Topic | Item | Checklist item description | Reported on Line | Completed |
| --- | --- | --- | --- | --- |
| Title | 1 | The diagnosis or intervention of primary focus followed by the words “case report” | Title page | Yes |
| Key words | 2 | 2 to 5 key words that identify diagnoses or interventions in this case report, including "case report" | Abstract | Yes (Premature infant, cyanotic apnea, infantile hemangioma, nasopharyngeal mass, neonatal airway obstruction, case report) |
| Abstract (no references) | 3a | Introduction: What is unique about this case and what does it add to the scientific literature | Abstract | Yes |
|  | 3b | Main symptoms and/or important clinical findings | Abstract | Yes |
|  | 3c | The main diagnoses, therapeutic interventions, and outcomes | Abstract | Yes |
|  | 3d | Conclusion—What is the main “take-away” lesson(s) from this case? | Abstract | Yes |
| Introduction | 4 | One or two paragraphs summarizing why this case is unique (may include references) | Introduction | Yes |
| Patient information | 5a | De-identified patient specific information | Case presentation | Yes |
|  | 5b | Primary concerns and symptoms of the patient | Case presentation | Yes ( Recurrent cyanotic apnea) |
|  | 5c | Medical, family, and psycho-social history including relevant genetic information | Case presentation | Yes (Prematurity, no other significant history) |
|  | 5d | Relevant past interventions with outcomes | Case presentation | Yes (Treated with pantoprazole initially for presumed reflux) |
| Clinical Findings | 6 | Describe significant physical examination (PE) and important clinical findings | Case presentation | Yes |
| Timeline | 7 | Historical and current information from this episode of care organized as a timeline | Case presentation | Yes |
| Diagnostic Assessment | 8a | Diagnostic testing (such as PE, laboratory testing, imaging, surveys) | Case presentation | Yes (bronchoscopy, CT scan) |
|  | 8b | Diagnostic challenges (such as access to testing, financial, or cultural) | Discussion | Yes (delayed diagnosis due to misattribution) |
|  | 8c | Diagnosis (including other diagnoses considered) | Case presentation | Yes (Nasopharyngeal infantile hemangioma) |
|  | 8d | Prognosis (such as staging in oncology) where applicable | Not applicable | favorable post-treatment |
| Therapeutic Intervention | 9a | Types of therapeutic intervention (such as pharmacologic, surgical, preventive, self-care) | Case presentation | Yes (surgery, oral prednisolone, propranolol) |
|  | 9b | Administration of therapeutic intervention (such as dosage, strength, duration) | Case presentation | Yes (drug type, oral, course) |
|  | 9c | Changes in therapeutic intervention (with rationale) | Case presentation | Yes (no changes indicated after initiation) |
| Follow-up and Outcomes | 10a | Clinician and patient-assessed outcomes (if available) | Case presentation | Yes (Follow-up imaging, symptom resolution) |
|  | 10b | Important follow-up diagnostic and other test result | Case presentation | Yes (no adverse effects reported, resolved symptoms, no recurrence) |
|  | 10c | Intervention adherence and tolerability (How was this assessed?) | Case presentation | Yes (no adverse effects reported, medication administered successfully) |
|  | 10d | Adverse and unanticipated events | Case presentation | Yes (none noted) |
| Discussion | 11a | A scientific discussion of the strengths AND limitations associated with this case report | Discussion | Yes |
|  | 11b | Discussion of the relevant medical literature with references | Discussion and Table of reviewed literature | Yes (Both in discussion and the table provided) |
|  | 11c | The scientific rationale for any conclusions (including assessment of possible causes) | Discussion, conclusion | Yes |
|  | 11d | The primary “take-away” lessons of this case report (without references) in a one paragraph conclusion | Discussion, conclusion | Yes |
| Patient Perspective | 12 | The patient should share their perspective in one to two paragraphs on the treatment(s) they received | Declarations | Yes |
| Informed Consent | 13 | Did the patient give informed consent? Please provide if requested | Declarations | Yes |
